# Supplementary figures and images for: Analysis of breast cancer survival in a northeastern Brazilian state based on prognostic factors: A retrospective cohort study
Source: PLoS One. 2022 Feb 3;17(2):e0263222. doi: 10.1371/journal.pone.0263222 (PMC8812889; doi:10.1371/journal.pone.0263222)

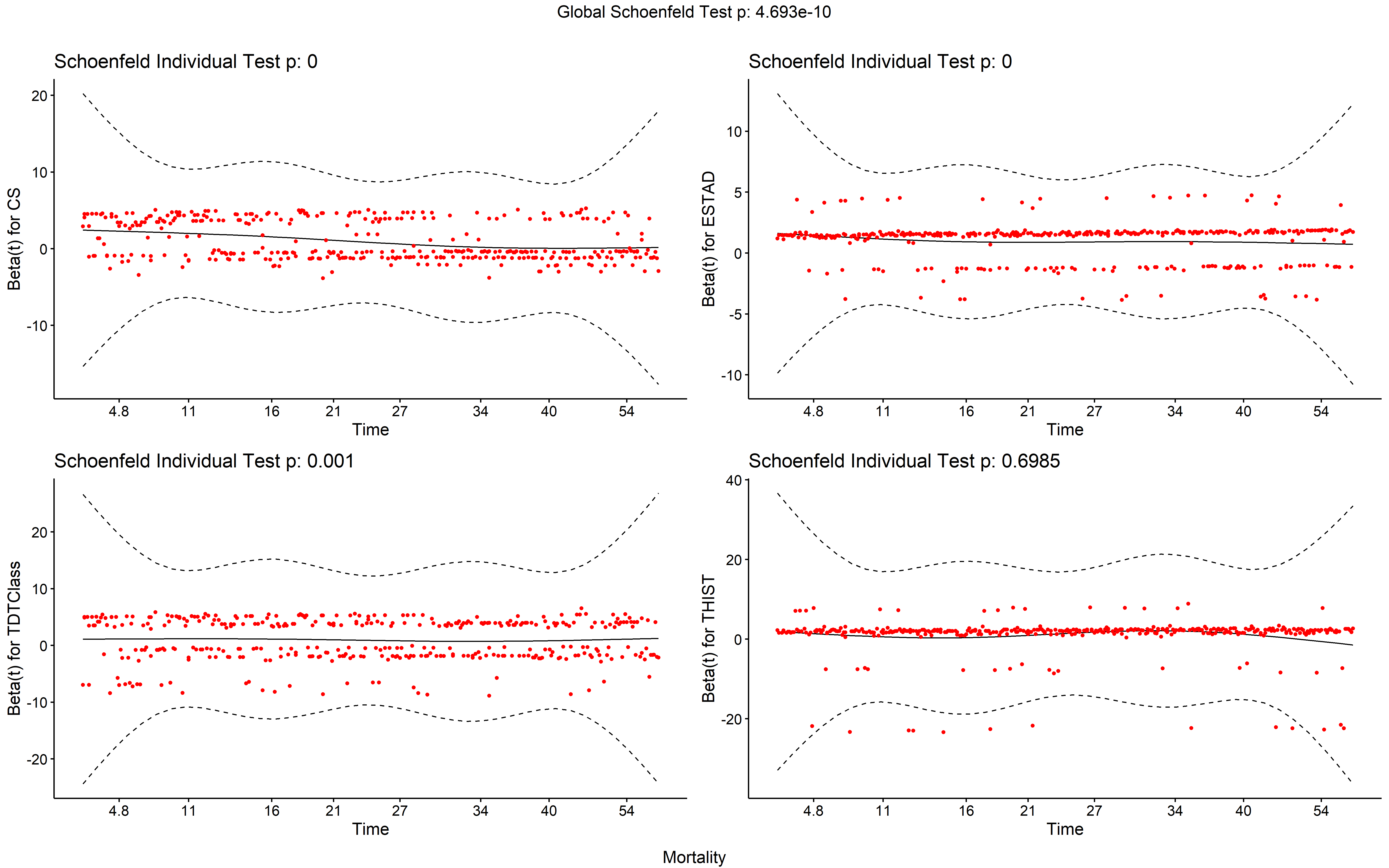

Supplement: S1 Fig — (TIFF) [file pone.0263222.s001.tiff]

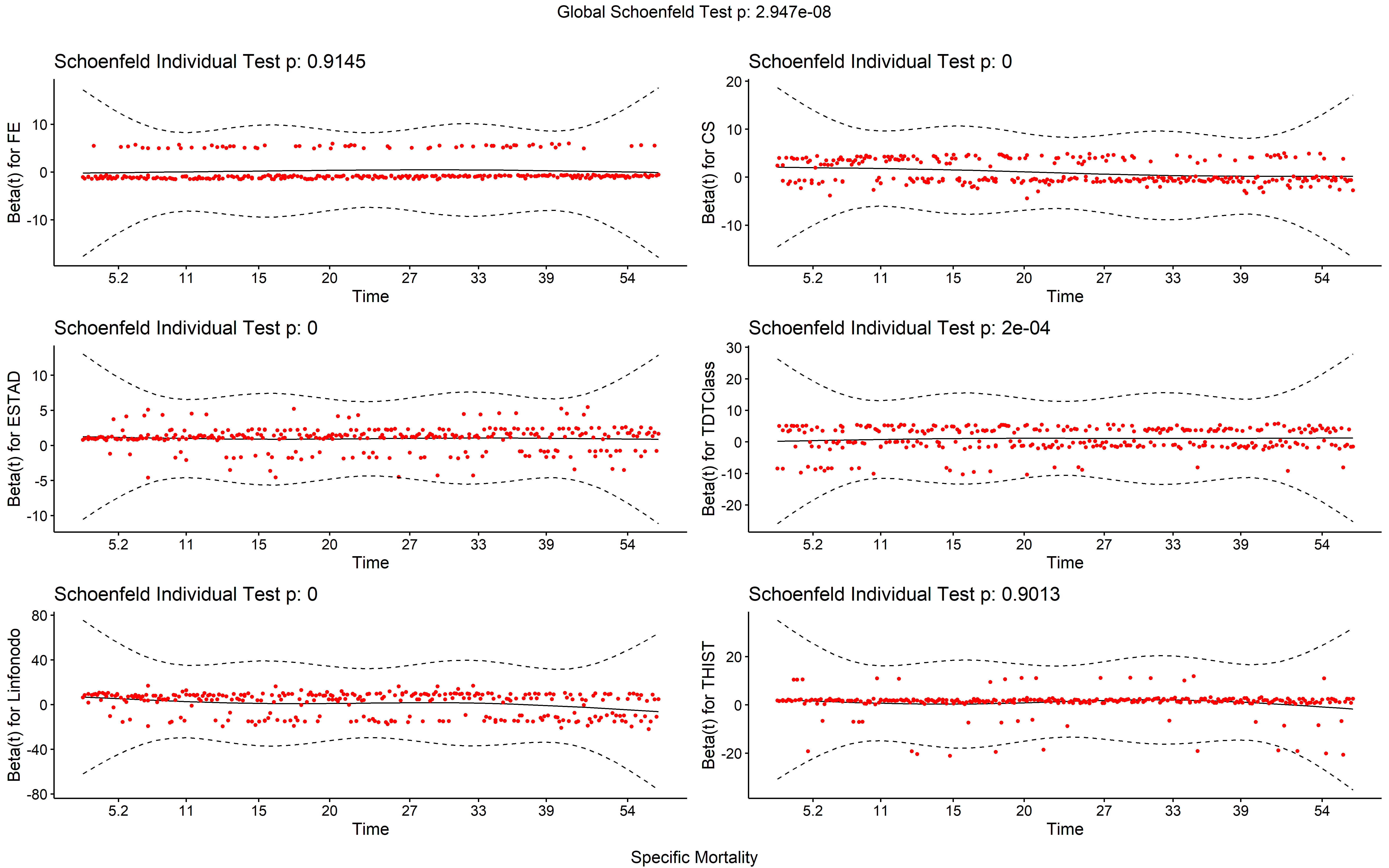

Supplement: S2 Fig — (TIFF) [file pone.0263222.s002.tiff]
