# Supplementary material for: Analysis of breast cancer survival in a northeastern Brazilian state based on prognostic factors: A retrospective cohort study
Source: PLoS One. 2022 Feb 3;17(2):e0263222. doi: 10.1371/journal.pone.0263222 (PMC8812889; doi:10.1371/journal.pone.0263222)
Supplement: Attachment — Submitted filename: Response to Reviewers - PONE-D-20-36356R3.docx [file pone.0263222.s004.docx]

Response to Reviewers

We had the final manuscript edited by Jeremy Allen, PhD, from Edanz (www.edanz.com/ac) and added acknowledgment.

Reviewer #1:

R. We thank you for all the comments addressed previously.

Reviewer #2: I am not convinced that the test of slope of schoenfeld residuals is too sensitive given the sample size (which isn't that large). Looking at the graphs of residuals, it appears that the top left graph may reflect non proportionality. Ideally, the authors would include a time dependent variable for that characterisitc. If this interaction term is signficant, that would be provide more evidence of non proportionality, and conveniently, address that at the same time.

R. After revision with the statistician, we agree with the reviewer’s opinion. So, we are including the lines “Even though the Schoenfield test rejects the hypothesis of hazard proportionality, Figs S3 and S4 show that hazards remain fairly constant throughout the follow-up period, except for the for the cancer-specific survival variables. Thus, to explain non proportionality, time-dependent variables were presented (Table 3)”; and also, are adding Table 3; as can be seen in the revised manuscript with track changes.
